# Comparative Study of Docosahexaenoic Acid with Different Molecular Forms for Promoting Apoptosis of the 95D Non-Small-Cell Lung Cancer Cells in a PPARγ-Dependent Manner

**DOI:** 10.3390/md20100599

**Published:** 2022-09-23

**Authors:** Hao Yue, Yingying Tian, Zifang Zhao, Yuying Bo, Yao Guo, Jingfeng Wang

**Affiliations:** 1 College of Food Science and Engineering, Ocean University of China, Qingdao 266003, China; 2 Marine Biomedical Research Institute of Qingdao, Qingdao 266071, China; 3 Hainan Huayan Collagen Technology Co., Ltd., Haikou 571000, China

**Keywords:** docosahexaenoic acid, different molecular forms, 95D non-small-cell lung cancer cell line, PPARγ, apoptosis

## Abstract

Cancer is a leading cause of death in worldwide. Growing evidence has shown that docosahexaenoic acid (DHA) has ameliorative effects on cancer. However, the effects of DHA-enriched phosphatidylcholine (DHA-PC) and efficacy differences between DHA-PC, DHA-triglyceride (DHA-TG), and DHA- ethyl esters (DHA-EE) on cancer cells had not been studied. In this study, 95D lung cancer cells in vitro were used to determine the effects and underlying mechanisms of DHA with different molecular forms. The results showed that DHA-PC and DHA-TG treatment significantly inhibited the growth of 95D cells by 53.7% and 33.8%, whereas DHA-EE had no significantly effect. Morphological analysis showed that DHA-PC and DHA-TG prompted promoted cell contraction, increased concentration of cell heterochromatin, vacuolization of cytoplasm, and edema of endoplasmic reticulum and mitochondria. TUNEL and AO/EB staining indicated that both DHA-PC and DHA-TG promoted cell apoptosis, in which DHA-PC performed better than DHA-TG. Mechanistically, DHA-PC and DHA-TG treatment up-regulated the PPARγ and RXRα signal, inhibited the expression of NF-κB and Bcl-2, and enhanced the expression of Bax and caspase-3, thereby promoting cell apoptosis. In conclusion, DHA-PC exerted superior effects to DHA-TG and DHA-EE in promoting apoptosis in 95D non-small-cell lung cancer cells. These data provide new evidence for the application of DHA in treatment of cancer.

## 1. Introduction

Cancer is a burdensome global health problem and leading cause of death for the middle-aged and elderly. According to statistics, more than 9.6 million people worldwide die from cancer each year, with an incidence rate of 1 in 6 [1]. Moreover, lung cancer has the highest incidence rate and mortality. Disappointingly, the reduced sensitivity of radiotherapy and chemotherapy and the propensity for recurrence and metastasis have made the efficacy of cancer drugs very limited, despite extensive drug discovery, development research, and efforts to improve treatment strategies [2]. Recently, the concept that dietary changes improved cancer treatment response has attracted more and more attention. For example, Kanarek et al. reported that dietary supplementation with histidine improved the sensitivity of methotrexate to cancer treatment [3]. Lambert et al. confirmed that dietary tocopherols significantly inhibited lung cancer growth in mice [4]. Therefore, the search for effective anti-cancer components from food sources has become a viable strategy for the treatment of cancer.

Peroxisome-proliferation-activated receptor gamma (PPARγ) is a member of the nuclear receptor superfamily, and is considered to play a critical regulatory role in cell differentiation, maintenance, and function [5]. In recent decades, substantial evidence indicates that the dysregulation of the PPARγ signal was linked to tumor development in the lungs, colon, and breast [6]. Preclinical studies have shown that PPARγ ligands (such as CB11, CB13 and PPZ023) could exert anti-tumor effects against a variety of other cancers [7]. Skelhorne-Gross et al. confirmed that PPARγ agonists can inhibit the growth of cancer cells in vitro, which was reversed by GW9662 (an inhibitor of PPARγ) [8]. Notably, PPARγ negatively regulates NF-κB expression by ligand-dependent transrepression, which was closely associated with cancer cell apoptosis [9]. Previous studies suggested that PPARγ agonists could directly interfere with the activation of NF-κB and inhibit cancer development [10]. Barkett et al. reported that NF-κB promoted the transcription of genes related with the anti-apoptosis [11]. Hafeez et al. confirmed that delphinidin treatment induced cell apoptosis via inhibiting the expression of NF-κB [12].

Docosahexaenoic acid (DHA) is a type of ω-3 long-chain polyunsaturated fatty acid, which is known to have a variety of nutritional and pharmacological effects [13]. It has been widely reported that DHA plays multi-functional roles in alleviating cancer progress [14]. In vitro experiments showed that treatment with DHA blocked cancer cell cycle progression. Cohort studies showed that a high intake of DHA significantly reduced the risk of breast cancer [15]. Du et al. reported that DHA exhibits synergistic therapeutic efficacy with cisplatin in the treatment of pancreatic cancer [16]. Notably, DHA mainly exists with different forms in a natural state, such as triacylglycerol (DHA-TG), phospholipids (DHA-PC), and ethyl esters (DHA-EE). Several research studies have shown that DHA-PC exhibit higher bioavailability due to the amphiphilic structure of the phospholipid molecules [17,18]. Recently, DHA-PC demonstrated superior anti-cancer activity in liver cancer [19]. However, the effects of DHA-PC, and efficacy differences between DHA-PC, DHA-triglyceride (DHA-TG), and DHA-ethyl esters (DHA-EE) on cancer cells had not been studied. Therefore, in the present study, we first compared the effects of DHA-PC, DHA-TG, and DHA-EE on the non-small-cell lung cancer cell line 95D and focused on its role in apoptosis. Further, the potential molecular mechanism was explored and verified.

## 2. Results

### 2.1. Effect of DHA with Different Molecular Forms on the Cell Viability of 95D Cells

**Figure 1:**
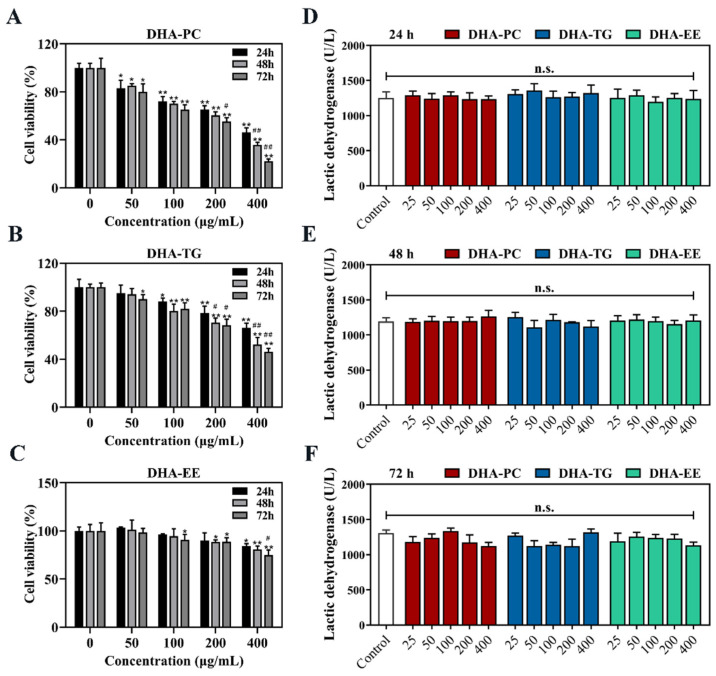
Effects of DHA with different molecular forms on the growth of 95D lung cancer cells. (**A**) Cell viability in DHA-PC group. (**B**) Cell viability in DHA-TG group. (**C**) Cell vi-ability in DHA-EE group. (**D**) LDH activity in 24 h. (**E**) LDH activity in 48 h. (**F**) LDH activity in 72 h. Data are presented as mean ± SD (n = 6). * *p* < 0.05, ** *p* < 0.01 versus control group. # *p* < 0.05, ## *p* < 0.01 versus 24 h. n.s., no significance. Abbreviations: lactic dehydrogenase (LDH).

Cancer cells are characterized by rapid proliferation rate and metastatic potential [20]. As shown in Figure 1A, compared to the control group, DHA-PC and DHA-TG treatment significant decreased the cell viability of 95D cells in a dose- and time-dependent manner (*p* < 0.01, 53.7% and 33.8% in 400 μg/mL, respectively). However, DHA-EE had a significant effect on the cell viability of 95D cells only at high concentrations (*p* < 0.05, 13.5% in 400 μg/mL). Furthermore, all treatment groups had no significant effect on LDH activity compared with the control group (Figure 1B, *p* > 0.05). These results indicated that DHA-PC and DHA-TG effectively inhibited the growth of 95D cells in a non-cytotoxic manner.

### 2.2. Effect of DHA with Different Molecular Forms on the Morphology of 95D Cells

**Figure 2:**
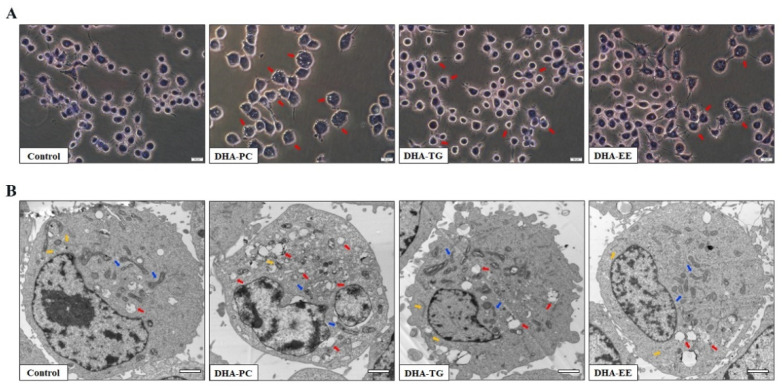
Effects of DHA with different molecular forms on the morphological changes in 95D lung cancer cells. (**A**) Hematoxylin staining. (**B**) Sub-microstructures observed by Transmission Electron Microscopy (magnification, ×10,000; scale bar: 2 μm; red arrow: cytoplasmic vacuole, yellow arrow: endoplasmic reticulum, blue arrow: mitochondria).

The morphological changes of the 95D cells were observed by hematoxylin staining and TEM. As shown in Figure 2A, different molecular forms of DHA (especially DHA-PC) treatment prompted severe morphological changes in 95D cells compared with the control group, including cell enlargement and rounding, blurred cell membrane borders, and intracellular vacuoles. Further, the sub-microstructural results showed that treatment with different molecular forms of DHA resulted in a concentration of cellular heterochromatin, severe vacuolization of the cytoplasm, and different degrees of edema in the endoplasmic reticulum and mitochondria (Figure 2B). Similarly, DHA-PC ameliorated microarchitectural degeneration more significantly than DHA-TG and DHA-EE. These results of morphological changes suggested that different molecular forms of DHA treatment may decrease the cell viability by inducing 95D cell apoptosis.

### 2.3. Effect of DHA with Different Molecular Forms on Apoptosis in 95D Cells

**Figure 3:**
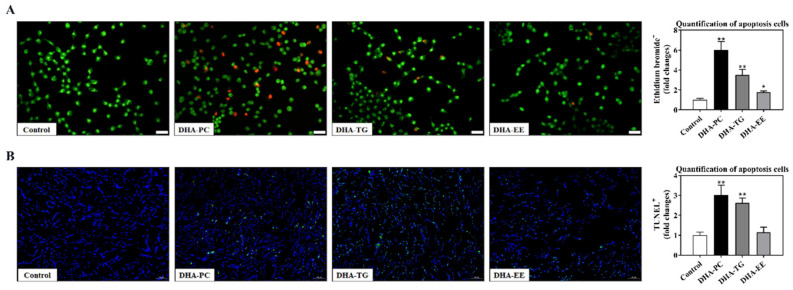
Effects of DHA with different molecular forms on apoptosis in 95D lung cancer cells. (**A**) Acridine orange/ethidium bromide staining (magnification, ×400; scale bar, 20 μm; Green fluorescence: normal cells, orange fluorescence: apoptotic cells). (**B**) Terminal deoxynucleotidyl transferase dUTP Nick-End Labeling staining. * *p* < 0.05, ** *p* < 0.01 versus control group.

AO/EB staining and TUNEL staining were performed to evaluate the effects of DHA with different molecular forms on apoptosis of 95D cells. As shown in Figure 3A, the apoptosis rate of 95D cells after DHA-PC and DHA-TG treatment were significantly higher than that of the control group, whereas there is no significant difference in DHA-EE. Furthermore, the TUNEL assay also revealed that DHA-PC and DHA-TG treatment significantly increased the number of apoptotic cells.

### 2.4. Effect of DHA with Different Molecular Forms on the PPARγ Expression in 95D Cells

**Figure 4:**
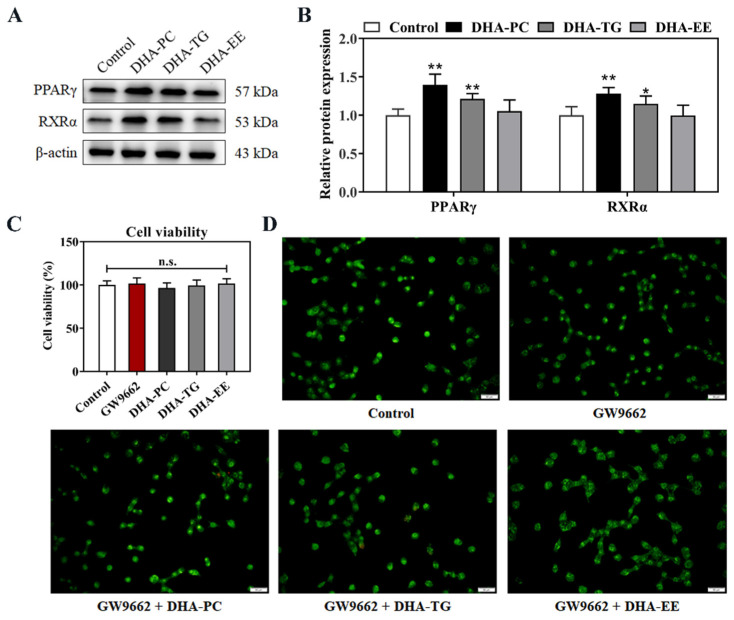
Effects of DHA with different molecular forms on the expression of PPARγ in 95D lung cancer cells (**A**) Western bolt analysis for PPARγ and RXRα protein expression. (**B**) Quantitative analysis of the PPARγ and RXRα protein expression levels. (**C**) Cell viability. (**D**) Acridine orange/ethidium bromide staining (magnification, ×400; scale bar, 20 μm; Green fluorescence: normal cells, orange fluorescence: apoptotic cells). Data are presented as mean ± SD (n = 6). * *p* < 0.05, ** *p* < 0.01 versus control group. n.s., no significance. Abbreviations: peroxisome-proliferation-activated receptor gamma (PPARγ), retinoid X receptor alpha (RXRα).

The retinoid X receptor alpha (RXRα) is a member of the nuclear receptor superfamily that regulates transcription of target genes through heterodimerization with PPARγ. PPARγ/RXRα signal has been proven to inhibit the growth of cancer cells and reduce tumor invasiveness in a variety of cancers [21]. As shown in Figure 4, DHA-PC and DHA-TG significantly enhanced the expression of PPARγ (*p* < 0.01, 39.5% and 21.4%, respectively,) and RXRα (*p* < 0.05, 27.9% and 15.2%, respectively) proteins. Further, PPARγ antagonist GW9662 abolished the apoptosis induced by DHA-PC and DHA-TG treatments (Figure 2C,D). These results indicated that DHA-PC and DHA-TG promoted the cell apoptosis in a PPARγ-dependent manner.

### 2.5. Effect of DHA with Different Molecular Forms on the PPARγ/NF-κB Signaling Pathway

**Figure 5:**
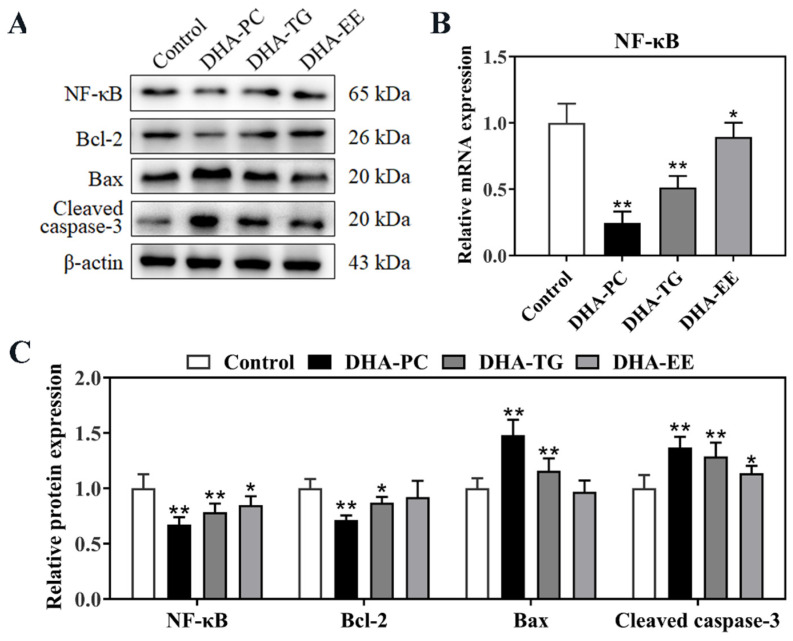
Effects of DHA with different molecular forms on the expression of PPARγ/NF-κB signaling pathway in 95D lung cancer cells (**A**) Western bolt analysis for NF-κB, Bcl-2, Bax, and cleaved caspase-3 protein expression. (**B**) Quantitative RT-PCR analysis of the mRNA expression levels of NF-κB. (**C**) Quantitative analysis of the NF-κB, Bcl-2, Bax, and cleaved caspase-3 protein expression levels. Data are presented as mean ± SD (n = 6). * *p* < 0.05, ** *p* < 0.01 versus control group. Abbreviations: nuclear transcription factor-κB (NF-κB), B-cell lymphoma-2 (Bcl-2), Bcl-2 associated X protein (Bax), cleaved cysteinyl aspartate specific proteinase-3 (Cleaved caspase-3).

PPARγ/RXRα has been proven to negatively regulate the NF-κB signal [22]. As shown in Figure 5A,B, the protein expression of NF-κB in 95D cells were significantly decreased in DHA-PC, DHA-TG, and DHA-EE groups by 32.8% (*p* < 0.01), 21.6% (*p* < 0.01), and 15.5% (*p* < 0.05) compared to the control group, respectively. The Bcl-2/Bax/caspase-3 signaling pathway is the downstream target of NF-κB, and plays an important role in cancer cells apoptosis. Figure 5A,C showed that the protein expression of Bax and cleaved caspase-3 were significantly increased, and the anti-apoptotic gene Bcl-2 was significantly decreased in the in DHA-PC and DHA-TG treatment groups. These results together indicated that DHA-PC and DHA-TG promoted the apoptosis in 95D cells via activating the PPARγ/NF-κB/Bcl-2 signaling pathway.

## 3. Discussion

A large body of evidence suggested that nutritional choices can influence the risk of developing certain malignancies [23]. Similarly, the daily diet could influence the progression of cancer, and about half of all cancer patients change their dietary habits in an effort to improve survival [24]. In recent decades, there is growing evidence that ω-3 polyunsaturated fatty acids play an active role in cancer treatment and prevention, in which, DHA has been proven to not only show anti-tumor activity, potentially as an effective adjuvant for cancer chemotherapy, but also to improve the secondary complications related to cancer (such as cachexia) [25]. In the present study, we demonstrated that DHA-PC significantly inhibited the growth of 95D non-small-cell lung cancer cells, which was superior to DHA-TG and DHA-EE. Furthermore, DHA with different molecular forms has no effects on LDH activity, indicating that the inhibited growth effects were mediated through the promotion of apoptosis. Mechanism studies reveal that DHA-PC, as an activator of PPARγ, promoted apoptosis by up regulating the PPARγ- mediated NF-κB/BCL-2 signaling pathway.

Nuclear receptors play critical roles in homeostasis [26]. As the representative member of the nuclear receptor superfamily, PPARγ exerts gene regulatory effects through ligand-dependent transactivation. It has been shown that PPARγ is highly expressed in most tumor cells, which is closely linked to cell cycle arrest, differentiation, and apoptosis [27]. Recent studies indicated that polyunsaturated fatty acids, thiazolidinedione, and some lipid-lowering drugs could enhance the transcriptional activity of PPARγ. Liu et al. found that DHA-PC inhibited angiogenesis through activating PPARγ in endothelial cells [28]. Ghnaimawi et al. confirmed that DHA treatment activated PPAR to promote the transdifferentiation of C2C12 cells into adipocyte phenotypes [29]. Similarly, the cytotoxicity of DHA on many tumor cells is related to the activation of PPARγ. Zand et al. revelated that DHA induced apoptosis in Reh cells by up-regulating the PPARγ signal, which reversed the antagonization of PPARγ with GW9662 [10]. Liu et al. found that DHA-PC suppressed Lewis lung cancer growth by activating PPARγ in mice [30]. In addition, the complete form of PPAR protein is a heterodimer composed of PPARγ and RXRα. In this study, our data clearly showed DHA-PC and DHA-TG treatment significantly upregulated the expression of PPARγ and RXRα, but DHA-EE had little effect.

The involvement of NF-κB in the development and progression of cancer has been widely reported [31]. Clinical evidence suggested that NF-κB directly regulates key gene expressions in cancer-related processes such as cancer cell proliferation, apoptosis, angiogenesis, and metastasis. Some studies indicated that apoptosis and cell arrest induced by PPARγ ligands may be mediated by the NF-κB-dependent pathway [32]. Silva-Gomez et al. confirmed that the preventitive effects of pirfenidone were mediated by the PPARγ/NF-κB signaling pathway. Notably, biochemical interaction studies showed that PPARγ bound with NF-κB prevents its translocation to the nucleus. The overexpression of PPARγ could promote the ubiquitination degradation of NF-κB, resulting in anticancer effects [33,34]. Lee et al. reported that ligand-activated PPARγ induced cell apoptosis by blocking the anti-apoptotic signaling of NF-κB [35]. Importantly, the anti-apoptotic activity of NF-κB is mediated through genes such as Bcl-2. Fahy et al. indicated that NF-κB inhibitor directly leads to the overexpression of Bcl-2 in a variety of malignant tumors. The same study reported that mutation of the NF-κB site decreased Bcl-2 promoter activity in all cell lines [36]. Li et al. found that siRNA silencing of NF-κB decreased the expression of Bcl-2 and Bax, thereby reducing cell apoptosis [37]. Consistent with the above studies, we found that DHA-PC and DHA-TG treatment inhibited NF expression by enhancing PPAR, significantly increased the expression of Bax and caspase3, and decreased the expression of Bcl-2. Furthermore, TUNEL, AOEB staining, and TEM results also confirmed that apoptosis was significantly increased in DHA-treated 95D cells.

Although there is sufficient evidence of the pharmacological role of DHA in cancer, clinical and epidemiological data do not seem to fully support this view. For example, Farrell et al. found that there was no significant relationship between DHA levels and risk for prostate cancer [38]. Importantly, dietary supplementation with different molecular forms of DHA has a huge efficiency gap in achieving intracellular pharmacological concentrations. Lawson et al. found that the absorption rates of triacylglycerol and ethyl ester DHA in vivo were only 57% and 21% [39]. Structurally, the ingestion of DHA undergoes both cell passive translocation and carrier-mediated transmembrane translocation, and the different molecular structure of DHA causes great differences in bioavailability and pharmacological activity [40]. Previous studies confirmed that phosphatidylcholine-bound substances more readily translocate through the cell membrane into the cell since the phospholipid has an amphiphilic molecular structure [41]. Kidd et al. reported that DHA-PC can be directly ingested by the cells in a binding form and decomposed into DHA and phosphatidylcholine, showing enhancing effects [42]. Zhang et al. reported that DHA-PC have a more significant effect on osteoporosis as compared with DHA-TG or DHA-EE [17]. Similarly, we found that DHA-PC with higher bioavailability has the most significant effect on apoptosis of 95D cells in this study, whereas DHA-EE has no effects. Notably, although DHA-EE has no significant effect on the expression of PPARγ, it significantly increased the expression of NF-κB and cleaved caspase-3. As a class of nuclear hormone receptor family, PPARγ has been proven to promote cell apoptosis through multiple pathways, such as NF-κB, PI3K, and MAPK, etc. We believe that the stronger apoptosis promoting effect of DHA-PC and DHA-TG on 95D non-small-cell lung cancer cells was mediated by PPARγ, whereas DHA-EE may have additional potential targets. The current study focuses on the effect of DHA with different molecular forms on the PPARγ-mediated NF-κB signal in 95D non-small-cell lung cancer cells apoptosis. On this basis, further studies will investigate in more detail PPARγ and downstream signals to better explain the positive role of different molecular forms of DHA in promoting cancer cell apoptosis.

## 4. Materials and Methods

### 4.1. Preparation of DHA

DHA-TG and DHA-EE were provided by Himega Biopharm Co., Ltd. (Yibin, China). DHA-PC was extracted following the described previously from squid roe (Zhoushan Fisheries Co., Ltd., Zhoushan, China). The accounted amount of DHA-PC was determined using an Agilent 7820 Gas Chromatograph with a flame-ionization detector. The purity of DHA-PC, DHA-TG, and DHA-EE were all higher than 90%.

### 4.2. Liposome Preparation

DHA liposomes samples were prepared according to the modified method of Hossain [19]. In brief, DHA and cholesterol (1:1) are dissolved in eggplant bottles containing a small amount of chloroform. Then, it was purged with nitrogen and treated with ultrasonic to form emulsion water suspension, and finally passed through 0.22 μM microporous membrane filtration to obtain liposomes.

### 4.3. Cell Culture and Viability Assay

The 95D cells were purchased from Shanghai Institutes for Biological Sciences (Shanghai, China) and cultured in an RPMI 1640 medium containing 10% fetal bovine serum and 0.1% penicillin-streptomycin at 37 °C with 5% CO_2_. The cell viability of 95D cells were evaluated by MTT method. Briefly, logarithmic growth phase 95D cells were grown in 96-well culture plates (2 × 10^3^ cells per plate) for 12 h and treated with different concentrations DHA samples for 24 h, 48 h, and 72 h. Then, cells were incubated with MTT solution (0.5 mg/mL) for 4 h and measured at 570 nm. The LDH contents of 95D cells were measured by commercial kits.

### 4.4. Hematoxylin and Acridine Orange/Ethidium Bromide (AO/EB) Staining

The 95D cells (3 × 10^3^ cells/per well) were inoculated with DHA samples (100 μg/mL) on 24-well plates for 24 h. The cells used for hematoxylin staining were washed twice in D-Hanks and then fixed in 95% ethanol for 20 min. Then, the cells were incubated with 0.5% hematoxylin staining solution for 5 min and washed with deionized water. The cells used for AO/EB staining were incubated with 10 μL/mL of AO (100 μg/mL) and EB (100 μg/mL) for 30 s, and washed with PBS. The morphology of cells was observed using an Olympus microscope (IX51, Tokyo, Japan).

### 4.5. Terminal Deoxynucleotidyl Transferase dUTP Nick-End Labeling (TUNEL) Staining

The 95D cells (3 × 10^3^ cells/per well) were inoculated with DHA samples (100 μg/mL) on 24-well plates for 24 h. The cells used for hematoxylin staining were washed twice in D-Hanks and then fixed in 95% ethanol for 20 min. Then, the cells were incubated with 80 μL TUNEL reaction solution for 90 min at 37 °C in a humidified dark chamber, Finally, the nuclei were stained with 10 μg/mL DAPI solution. The morphology of cells was observed using an Olympus microscope (IX51, Japan).

### 4.6. Transmission Electron Microscopy (TEM)

The 95D cells treatment with DHA samples (100 μg/mL) were inoculated for 24 h, and fixed with 2.5% glutaraldehyde. After ethanol dehydration, stained with uranyl acetate and lead citrate, the fixed cells were analyzed by a JEM-1200EX transmission electron microscope.

### 4.7. Quantitative Real-Time PCR Analysis

The 95D cells (1 × 10^6^ cells/per well, 6-cell plates) were incubated with DHA samples (100 μg/mL) for 24 h, and total was extracted RNA by the RNeasy Mini Kit. The amplification conditions were as hereunder mentioned: pre-denatured at 95 °C for 10 min, denatured at 95 °C for 15 s, annealed at 60 °C for 30 s, and extended at 72 °C for 45 s of 40 cycles.

### 4.8. Western Blot Analysis

The 95D cells (1 × 10^6^ cells/per well, 6-cell plates) were incubated with DHA samples (100 μg/mL) for 24 h, and lysed using RIPA buffer to obtain total protein. The protein samples were separated in SDS-PAGE by electrophoresis and transferred to a PVDF membrane. Subsequently, the membrane was blocked with 5% bovine serum albumin for 2 h at room temperature. After that, the membrane was incubated overnight at 4 °C with primary antibodies (Cell Signaling Technologies, 1:1000) and then incubated with the secondary antibody (Proteintech, 1:4000) for 2 h. The protein results were detected by an enhanced chemiluminescence method using the Bio-Spectrum Gel Imaging System (UVP, Upland, CA, USA).

### 4.9. Statistical Analysis

All data were statistically analyzed used SPSS version 22.0 by One-way analysis of variance (ANOVA) with Turkey’s test. Statistical difference was considered significant at *p* < 0.05.

## 5. Conclusions

In conclusion, the current study demonstrated for the first time that the effect of DHA on non-small-cell lung cancer cells is related to its molecular form. Moreover, DHA-PC was a superior choice for beneficial effects on promoting cells apoptotis over DHA-TG and DHA-EE, by up-regulating of the NF-κB/BCL-2 signaling pathway in a PPARγ-dependent manner. We believe these findings provide new evidence for the treatment of cancer via dietary intervention and the development of functional dietary supplements.

## Data Availability

Not applicable.
